# Benchmarking of bioinformatics tools for NGS-based microRNA profiling with RT-qPCR method

**DOI:** 10.1007/s10142-023-01276-w

**Published:** 2023-11-30

**Authors:** Klaudia Pawlina-Tyszko, Tomasz Szmatoła

**Affiliations:** 1 Department of Animal Molecular Biology, National Research Institute of Animal Production, Krakowska 1 st., 32-083 Balice, Poland; 2 Center for Experimental and Innovative Medicine, University of Agriculture in Krakow, Redzina 1c, 30-248 Krakow, Poland

**Keywords:** MicroRNA, qPCR, NGS, Bioinformatics

## Abstract

MicroRNAs are vital gene expression regulators, extensively studied worldwide. The large-scale characterization of miRNAomes is possible using next-generation sequencing (NGS). This technology offers great opportunities, but these cannot be fully exploited without proper and comprehensive bioinformatics analysis. This may be achieved by the use of reliable dedicated software; however, different programs may generate divergent results, leading to additional discrepancies. Thus, the aim of this study was to compare three bioinformatic algorithms dedicated to NGS-based microRNA profiling and validate them using an alternative method, namely RT-qPCR. The comparison analysis revealed differences in the number and sets of identified miRNAs. The qPCR confirmed the expression of the investigated microRNAs. The correlation analysis of NGS and qPCR measurements showed strong and significant coefficients for a subset of the tested miRNAs, including those detected by all three algorithms. Single miRNA variants (isomiRs) showed different levels of correlation with the qPCR data. The obtained results revealed the good performance of all tested programs, despite the observed differences. Moreover, they implied that some specific miRNAs may be differentially estimated using NGS technology and the qPCR method, regardless of the used bioinformatics software. These discrepancies may stem from many factors, including the composition of the isomiR profile, their abundance, length, and investigated species. In conclusion, in this study, we shed light on the bioinformatics aspects of miRNAome profiling, elucidating its complexity and pinpointing potential features influencing validation. Thus, qPCR validation results should be open to interpretation when not fully concordant with NGS results until further, additional analyses are conducted.

**Supplementary Information:**

The online version contains supplementary material available at 10.1007/s10142-023-01276-w.

## Introduction

MicroRNAs are of interest to many research groups because of their extensive regulatory interaction networks and potential to serve as biomarkers (Condrat et al. 2020). Although they do not code for proteins, they orchestrate gene expression regulation by binding to mRNAs and preventing further protein synthesis. Thanks to the ability to bind to the transcripts of many genes, miRNAs are indirectly involved in numerous cellular processes, such as apoptosis or proliferation, which are crucial for the functioning of cells, tissues, and organisms (Fabian et al. 2010; Filipowicz et al. 2008; He and Hannon 2004). The development of modern technologies has allowed for in-depth analysis of microRNA functioning, including large-scale profiling of entire miRNAomes in different species and tissues, both in physiological and pathological conditions (Chen et al. 2014; Chirayil et al. 2018; Lagos-Quintana et al. 2002; Pawlina et al. 2017; Ropka-Molik et al. 2018).

Currently, the greatest analytical possibilities are offered by high-throughput next-generation sequencing (NGS) technologies. These enable the generation of a large amount of sequence data in a relatively short time, without the need for prior knowledge of the analysed sequences. Therefore, they provide not only the possibility of identifying new microRNAs, which is extremely important for expanding the state of knowledge and better characterisation of the examined tissue and species, but also allow for large-scale expression analysis. One of the available NGS technologies is provided by Illumina, called sequencing by synthesis (SBS). It supports massively parallel base-by-base sequencing using fluorescently labelled dNTPs, which are incorporated into growing DNA strands (Bentley et al. 2008).

The use of next-generation sequencing to identify miRNAs not only offers a wide range of analytical possibilities but also entails certain technical requirements, including adequate computer and human resources. Knowledge of the biology of these sequences and bioinformatics is also required. The large number of available programs and ready-made scripts for the identification and analysis of microRNA expression using various analytical algorithms offer a wide range of choices (Lukasik et al. 2016), but can also lead to additional discrepancies in the obtained results. These programs are characterised by a different analytical approach, a different form of interface, often not very user-friendly, and, most importantly, varied sensitivity and specificity. This is directly reflected in the number of identified sequences and the percentage of false positive and negative results.

Therefore, choosing the most suitable program to analyse a given type of data can often involve difficulties. The main and superior criterion is obtaining the most reliable results. In addition, however, a user may be also interested in the scope of analyses offered within a given program and, from a technical point of view, hardware requirements, the speed of calculations, and the accessibility of the form of generated results. Therefore, the selection of the optimal program may increase the credibility and comparability with other results available in the world literature as well as the repeatability of obtained results.

Thus, the aim of this study was to compare the results generated by three bioinformatic programs dedicated to microRNA sequence identification based on NGS data, followed by empirical validation of chosen miRNA sequences with the RT-qPCR technique, which is very often the method of choice for the NGS data validation (Everaert et al. 2017; Gurgul et al. 2017; Pawlina-Tyszko et al. 2020).

## Material and methods

### Comparative bioinformatics analysis

In the first stage, the results of next-generation sequencing of microRNA libraries obtained in the previous research projects (GEO accession numbers: GSE87901, GSE148302, GSE231434) (Niwińska et al. 2022; Pawlina et al. 2017; Pawlina-Tyszko et al. 2020) were subjected to quality analysis in order to select the best, most representative samples with phred quality > 20, using the FastQC program (Andrews 2010). Low-quality reads and adapter trimming as well as length filtering (18–25 nt) were carried out with the Trimmomatic software (Bolger et al. 2014). Then, based on the database of available software for microRNA analysis—Tools4miRs (Lukasik et al. 2016)—three programs suitable for known miRNA identification and prediction of novel sequences were selected. The main criterion was their common usage, according to the number of their citations in the world literature. An additional selection criterion was the long-term support of the authors, which ensures the development of the software and performance improvements, as well as bug fixes. The miRNA analysis was performed using the latest version of the microRNA sequence reference database miRBase 22.1 (Griffiths-Jones et al. 2008; Kozomara and Griffiths-Jones 2011) and the same reference genome for each tool (EquCab2.0, Sscrofa11.1, Bos_taurus_UMD_3.1). Default analysis settings were used along with the minimum number of reads set to 2. The obtained results were compared in terms of the number of known and new microRNAs identified, the calculation time, and hardware requirements.

Then, 21 microRNAs were selected for further validation using the qRT-PCR method in the next step. Because of differences in the number of identified microRNAs between individual programs, the selection of microRNAs for validation was dictated by an attempt to reflect the observed proportions as best as possible. Therefore, 11 miRNAs identified by miRDeep2 software were selected, including four known and seven new ones, as this program predicted the greatest number of novel sequences among the three programs analysed. Seven miRNAs profiled by sRNAtoolbox-sRNAbench were chosen for validation, including five known and two new ones, as this program identified the greatest number of known microRNAs of the three programs tested. Seven microRNAs identified by UEA sRNA Workbench were selected, including three known and four novel ones, as this software identified similar numbers of known and new microRNAs, intermediate compared to the other two programs. Moreover, the selection criteria included the priority for miRNAs with a read count ≥ 50, identified uniquely by one tool and having positive scores assigned by miRDeep2 and UEA sRNA Workbench. In case there were no high-quality sequences identified solely by one tool, alternative miRNAs detected by two programs were selected. The chosen miRNAs differentially identified by the tested programs were subjected to a pathway analysis using miRNet 2.0 analytics platform (Chang et al. 2020). Moreover, four miRNAs identified by all three programs were chosen as positive controls (miR-375, miR-215-5p, miR-708-5p, miR-381-3p).

### Validation with RT-qPCR

RNA samples selected in the previous step were reverse transcribed using the miRCURY LNA RT kit (Qiagen) according to the manufacturer’s protocol. qPCR was performed using the miRCURY LNA SYBR Green PCR Kit (Qiagen) and commercially available and custom-made primers (Online Resource 1) specific for selected microRNAs (miRCURY LNA miRNA PCR Assay; Qiagen), according to the standard protocol. Each reaction was performed in duplicate, on a Quant Studio 7 Flex instrument (Thermo Fisher Scientific). For each pair of primers, a negative control was included without the addition of a template (NTC). The melt curve analysis, including the additional analysis of ambiguous results using uMELT Quartz software (Dwight et al. 2011), was performed to confirm the specificity of the primers used. In order to assess the efficiency of the conducted reactions, the standard curve method was used for each pair of primers. U6 snRNA and 5S rRNA were used as endogenous controls for expression normalisation. In addition, each sample was run with primers specific for cel-miR-39-3p, which is not expressed in mammals, to further confirm the correct performance of the reaction kit used and other primers. The obtained results were analysed using the ΔΔCt calculation method (Pfaffl 2001) to determine the expression level relative to the endogenous control. Pearson correlation coefficients between the NGS and qPCR results were calculated with the devtools R package (Wickham et al. 2022).

## Results

### Comparative bioinformatics analysis

The initial analysis of the miRNA-seq data from the previous projects (Niwińska et al. 2022; Pawlina et al. 2017; Pawlina-Tyszko et al. 2020) allowed us to select a total of 30 samples of the highest quality: 10 from horse skin tissue, 10 from pig brain tissue, and 10 from cow mammary gland tissue. Then, three common, renowned miRNA calling programs were chosen using Tools4miRs database (Lukasik et al. 2016), namely miRDeep2 (v2.0.1.2) introduced in 2012 (Friedländer et al. 2012), sRNAtoolbox-sRNAbench introduced in 2014 (formerly miRanalyzer) (Aparicio-Puerta et al. 2019, 2022; Barturen et al. 2014), and UEA sRNA Workbench (v4.7) introduced in 2012 (Stocks et al. 2012, 2018).

These programs differ in their interface and the requirement of user advancement. miRDeep2 is a software package written in Perl programming language. It allows the identification and profiling of known miRNA sequences as well as the prediction of potentially new miRNAs in animals, based on deep sequencing data (Friedländer et al. 2012). It is a command-line package, which requires some knowledge on running this type of software. Moreover, basic bioinformatics knowledge is needed to pre-process sequences and set miRNA identification parameters. The analysis using this program takes on average several dozen minutes per sample depending on the available hardware.

sRNAtoolbox-sRNAbench allows microRNA profiling in multiple animal and plant species. It identifies known miRNA sequences, predicts novel miRNAs, and characterizes the expression profile of miRNA variants—isomiRs. It is available as a web tool, standalone version, and Docker container (Aparicio-Puerta et al. 2019, 2022; Barturen et al. 2014). The online version of sRNAtoolbox-sRNAbench does not require specialized IT knowledge; however, basic knowledge on trimming, quality and mapping of NGS reads is required. Since it is based on an external server, it is independent of the user’s hardware resources, and the analysis takes a few minutes per sample.

The UEA sRNA workbench is a downloadable software package. It allows complete analysis of single or multiple small RNA datasets from both plants and animals, profiling small RNA expression patterns and identifying novel microRNAs. It can be run on Windows, Linux, and MAC OS PCs with a java plugin (Stocks et al. 2012, 2018). Basic knowledge on microRNA characteristics and pre-processing of NGS results is required to prepare input sequences and set parameters. The analysis takes several minutes per sample if minimal recommended hardware requirements are met.

The number and type of generated output files differed between the tested programs. UEA sRNA Workbench generated a csv file with reads processing statistics and a csv file with details on identified sequences. Graphic files showing the length distribution of the identified microRNAs (.png) and secondary structures of their precursor sequences (.jpg) were also created (Online Resources 2 and 3). The miRDeep2 output embraced the main result table in csv, html, and bed formats, several run logs and statistics, as well as several separate files with details on the identified known and novel miRNAs in bed and fa formats. The identified stem-loop sequences with their characteristics and mapping details were graphically visualised in pdf format (Online Resource 4). sRNAtoolbox-sRNAbench generated several dozen output files including statistics and details on identified hairpins, known and novel mature sequences, and sequence variants (isomiRs), in txt and fa formats. Several graphs visualising, e.g. top microRNAs, mapping stats, and mapped reads, were in png format (Online Resources 5 and 6).

**Fig. 1:**
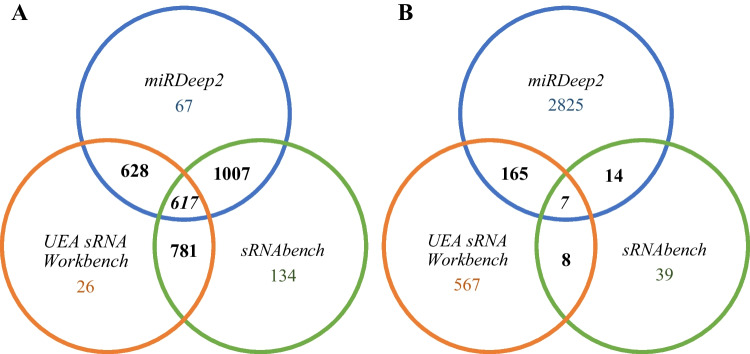
Venn diagram showing the number of identified known (**A**) and predicted novel (**B**) microRNAs, unique for each software and the common ones

Further analysis showed that UEA sRNA Workbench identified on average 181 known microRNAs per sample, followed by miRDeep2 (280) and sRNAtoolbox-sRNAbench (397). The number of predicted novel sequences ranged from 6 new miRNAs per sample on average (sRNAtoolbox-sRNAbench) through 131 (UEA sRNA Workbench) to 212 (miRDeep2). This was also reflected in the number of known and novel microRNAs identified for all samples tested (Table 1). When it comes to the software-unique sequences, UEA sRNA Workbench profiled 26 known and 567 novel miRNAs; miRDeep2 67 known and 2825 new sequences; while sRNAtoolbox-sRNAbench 134 known and 39 novel microRNAs (Table 1). The number of known miRNAs common for all three analysed tools amounted to 617, whereas the number of common novel microRNAs was equal to seven. Detailed data on the number of miRNAs common for two out of three programs are shown in Table 1 and Fig. 1.

**Table 1:**
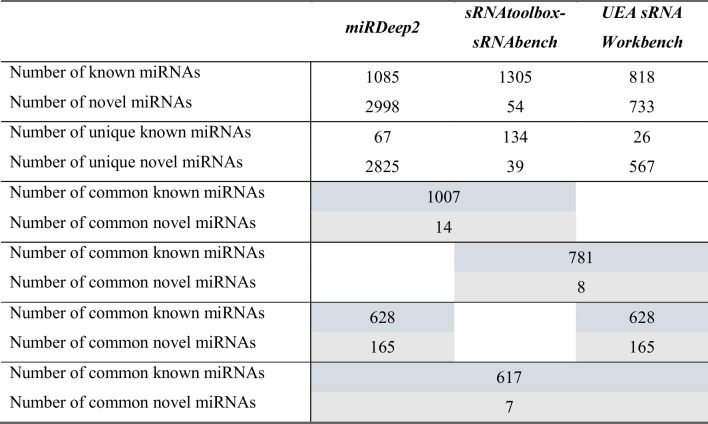
Data on the number of known and new microRNAs identified by the analysed programs in the 30 tested samples

The given values are for microRNAs meeting the criterion of the minimum number of two reads. The coloured blocks indicate data common for indicated algorithms

### qPCR validation results

Out of the 21 analysed microRNAs, bta-miR-574-specific primers were excluded from further testing since the reaction efficiency and NTC results were incorrect. The analysis of the melting curve of the other primers revealed high specificity, as one product was obtained for each of them. The negative controls used, i.e. negative without template (NTC) and cel-miR-39-3p, did not give detectable reaction products. The final analysis of the relative expression level showed the expression of all tested microRNAs, including the positive controls (Table 2 and Online Resource 7).

**Table 2:** Table showing details of the qPCR-validated microRNAs. Information is provided for each of the analysed program whether a tested microRNA was identified, and if yes, whether as known (xxx-miR-yyy) or new

No.	miRCURY PCR assay name	miRBase miRNA name			qPCR validation result (mean relative expression)
		*miRDeep2*	*sRNAtoolbox-sRNAbench*	*UEA sRNA workbench*	
1.	Custom 1	New	NI	NI	Confirmed (5.95)
2.	hsa-miR-375	New	NI	New	Confirmed (107.79)
3.	Custom 13	NI	New	New	Confirmed (8.63)
4.	hsa-let-7b-5p	NI	New	NI	Confirmed (6.69)
5.	hsa-miR-100-5p	bta-miR-100	bta-miR-100	NI	Confirmed (1.94)
6.	bta-miR-3432a	bta-miR-3432a	NI	bta-miR-3432a	Confirmed (4.29)
7.	hsa-miR-376c-3p	ssc-miR-376c	NI	NI	Confirmed (9.67)
8.	hsa-miR-223-3p	NI	bta-miR-223	bta-miR-223	Confirmed (3.02)
9.	hsa-miR-30c-5p	NI	bta-miR-30c	NI	Confirmed (1.81)
10.	Custom 31	NI	NI	New	Confirmed (4.41)
11.	hsa-miR-574-5p	NI	NI	bta-miR-574	Rejected because of incorrect reaction parameters
12.	cfa-miR-485	NI	NI	New	Confirmed (9.20)
13.	hsa-miR-329-3p	bta-miR-329b	NI	NI	Confirmed (2.73)
14.	tgu-miR-30b-3p	NI	NI	bta-miR-30a-3p	Confirmed (2.89)
15.	hsa-miR-433-3p	NI	bta-miR-433	NI	Confirmed (2.78)
16.	hsa-miR-451a	NI	bta-miR-451	NI	Confirmed (4.75)
17.	hsa-miR-1264	New	NI	NI	Confirmed (13.66)
18.	oar-miR-3959-5p	New	NI	NI	Confirmed (4.04)
19.	hsa-miR-346	New	NI	NI	Confirmed (7.04)
20.	Custom 47	New	NI	NI	Confirmed (2.09)
21.	bta-miR-211	New	NI	NI	Confirmed (10.17)
Negative control	cel-miR-39-3p	NI	NI	NI	Confirmed (0)
Positive control	hsa-miR-375	bta, ssc-miR-375	bta, ssc-miR-375	bta, ssc-miR-375	Confirmed (8.84)
Positive control	hsa-miR-215-5p	bta-miR-215	bta-miR-215	bta-miR-215	Confirmed (2.24)
Positive control	hsa-miR-708-5p	bta-miR-708 ssc-miR-708-5p	bta-miR-708 ssc-miR-708-5p	bta-miR-708 ssc-miR-708-5p	Confirmed (5.32)
Positive control	ssc-miR-381-3p	ssc-miR-381-3p	ssc-miR-381-3p	ssc-miR-381-3p	Confirmed (3.85)

*NI*, not identified; *Bta*, *Bos taurus*; *ssc*, *Sus scrofa*; *cel*, *Caenorhabditis elegans*; *hsa*, *Homo sapiens*; *tgu*, *Taeniopygia guttata*; *cfa*, *Canis familiaris*; *oar*, *Ovis aries*

In summary, all the selected 11 miRNAs identified by miRDeep2, seven determined by sRNAtoolbox-sRNAbench, and seven identified by UEA sRNA Workbench were confirmed by qPCR (Table 3). The KEGG pathway analysis allowed identification of 66 significantly enriched pathways (FDR ≤ 0.1) for miRDeep2 (including 10 unique ones), 89 for sRNAtoolbox-sRNAbench (27 unique), and 54 for UEA sRNA Workbench (3 unique) (Online Resource 8).

**Table 3:** Summary table of the RT-qPCR validation results

	*miRDeep2*	*sRNAtoolbox-sRNAbench*	*UEA sRNA workbench*
Number of validated miRNAs	11	7	7
Number of confirmed miRNAs	11	7	7
Number of validated known miRNAs	4	5	3
Number of confirmed known miRNAs	4	5	3
Number of validated new miRNAs	7	2	4
Number of confirmed new miRNAs	7	2	4

In the next step, we calculated correlation coefficients for the investigated miRNAs between the qPCR results and read counts outputted by each of the programs to assess the significance of different methods of reporting the miRNA read number. Moreover, since custom-made primers were used for some miRNAs, we performed additional reactions with alternative custom 31 and custom 47 assays. This approach was applied to gain insight into the potential influence of qPCR primers and assay design on the validation results.

For miRDeep2, four strong and significant correlations (*p*-value ≤ 0.05) were identified, namely for two investigated miRNAs, hsa-miR-375 (*R* = 0.95) and bta-miR-3432a (*R* = 0.92); and for two positive controls, hsa-miR-375 (cow samples; *R* = 0.74) and hsa-miR-708-5p (cow samples; *R* = 0.86). The use of the alternative custom 47 assay caused a substantial increase in the correlation coefficient from 0.09 to 0.59, and a decrease of *p*-value from 0.79 to 0.07. Three significant correlations (*p*-value ≤ 0.05) were revealed for sRNAtoolbox-sRNAbench: one strong for the examined hsa-miR-451a (*R* = 0.91) and two for the positive controls hsa-miR-375 (cow samples; *R* = 0.67) and hsa-miR-708-5p (cow samples; *R* = 0.92). Among the tested miRNAs identified by UEA sRNA workbench, three showed the moderate correlation level (*p*-value ≤ 0.05): hsa-miR-223-3p (*R* = 0.65), custom 31 (*R* = 0.67), and cfa-miR-485 (*R* = 0.68), while another three were strongly correlated (*p*-value ≤ 0.05): hsa-miR-375 (*R* = 0.95), bta-miR-3432a (*R* = 0.97), and tgu-miR-30b-3p (*R* = 0.78). For the alternative custom 31 assay, a decrease in the correlation coefficient was observed from 0.67 to − 0.42 accompanied by the loss of statistical significance (*p*-value = 0.27). Of the positive controls, the following two were moderately correlated (*p*-value ≤ 0.05): hsa-miR-375 (cow samples, *R* = 0.63) and hsa-miR-375 (pig samples, *R* = 0.66). When it comes to miRNAs called by two programs, their correlation coefficients and *p*-values were similar (Table 4).

**Table 4:** Data on the results of the correlation analysis between the qPCR and miRNA-seq results for the investigated assays

miRCURY PCR assay name	Correlation coefficient (*p*-value)				Exact isomiR abundance [%]
	*miRDeep2*	*sRNAtoolbox-sRNAbench*	*UEA sRNA workbench*	Assay exact isomiR	
Custom 1	− 0.07 (0.85)	-	-	-	-
hsa-miR-375	0.95 (0.05)	-	0.95 (0.05)	-	-
custom 13	-	0.59 (0.08)	0.59 (0.07)	-	-
hsa-let-7b-5p	-	0.45 (0.45)	-	-	-
hsa-miR-100-5p	0.33 (0.35)	0.42 (0.22)	-	0.58 (0.08)	0.07
bta-miR-3432a	0.92 (1.56E− 04)	-	0.97 (1.92E− 6)	-	-
hsa-miR-376c-3p	0.01 (0.98)	-	-	-	-
hsa-miR-223-3p	-	0.61 (0.06)	0.65 (0.04)	0.58 (0.07)	6.38
hsa-miR-30c-5p	-	0.24 (0.49)	-	0.10 (0.77)	1.94
custom 31	-	-	0.67 (0.05)	-	-
custom 31 v2	-	-	− 0.42 (0.27)	-	-
cfa-miR-485	-	-	0.68 (0.03)	-	-
hsa-miR-329-3p	0.19 (0.59)	-	-	-	-
tgu-miR-30b-3p	-	-	0.78 (8.07E−03)	-	-
hsa-miR-433-3p	-	0.59 (0.07)	-	0.69 (0.03)	0.20
hsa-miR-451a	-	0.91 (2.18E−04)	-	− 0.09 (0.79)	0.05
hsa-miR-1264	0.14 (0.69)	-	-	-	-
oar-miR-3959-5p	0.51 (0.19)	-	-	-	-
hsa-miR-346	− 0.23 (0.53)	-	-	-	-
custom 47	0.09 (0.79)	-	-	-	-
custom 47 v2	0.59 (0.07)	-	-	-	-
Positive controls					
hsa-miR-375 cow samples	0.74 (0.02)	0.67 (0.05)	0.63 (0.05)	0.56 (0.12)	26.38
hsa-miR-375 pig samples	0.59 (0.07)	0.59 (0.07)	0.66 (0.04)	0.60 (0.07)	18.74
hsa-miR-215-5p cow samples	0.41 (0.24)	0.35 (0.32)	0.22 (0.53)	− 0.17 (0.65)	0.07
hsa-miR-708-5p cow samples	0.86 (1.59E−03)	0.92 (1.63E−04)	0.01 (0.97)	0.89 (5.67E−04)	0.58
hsa-miR-708-5p pig samples	− 0.51 (0.13)	− 0.31 (0.38)	− 0.43 (0.21)	− 0.11 (0.75)	0.69
ssc-miR-381-3p pig samples	0.06 (0.86)	0.17 (0.65)	− 0.38 (0.27)	− 0.35 (0.32)	0.02

**Fig. 2:**
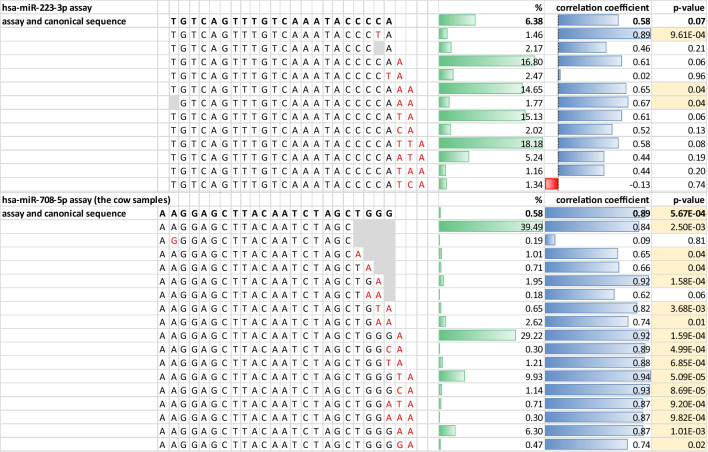
Graphic representation of results of the single isomiR correlation analysis for hsa-miR-223-3p and hsa-miR-708-5p assays. The assay and canonical miRNA sequences are bolded, while isomiR sequences are given below. Red nucleotides denote those different compared to the canonical sequence. Grey blocks represent the lack of a nucleotide compared to the canonical sequence. “%” stands for the share of a given variant in the whole miRNA read count. Yellow coloured cells highlight *p*-value ≤ 0.05

In the case of known miRNAs which were annotated by sRNAtoolbox-sRNAbench, the calculations were also performed for separate isomiRs, including the isomiR with the same sequence as the used assay, to elucidate the influence of different miRNA sequences and length variants on qPCR validation results. As a result, one statistically significant (*p*-value ≤ 0.05) moderate correlation for the exact isomiR of hsa-miR-433-3p (*R* = 0.69) and one strong correlation for the exact isomiR of the positive control hsa-miR-708-5p (*R* = 0.89) were revealed. Correlation coefficients and statistical significance increased for two out of 11 miRNAs for which this approach was applied (hsa-miR-100-5p, hsa-miR-433-3p) (Table 4). The results obtained for the other isomiRs showed different levels of correlation and significance, including sequences differing in one nucleotide and those with different expression levels (Fig. 2 and Online Resource 9).

## Discussion

MicroRNA expression profiling is a commonly used analysis in many types of experiment. It provides valuable information on the functioning of the cellular machinery of various tissues and species, as well as its disorders in various disease states. In-depth characterization of the miRNA profile is enabled by next-generation sequencing technology. There is a large number of miRNA identification programs allowing for the annotation of already known microRNAs and prediction of potentially new ones. Since they are based on different algorithms and may apply different identification criteria, they may give different results. Therefore, in this study, we empirically evaluated the performance of three programs for microRNA analysis with the RT-qPCR method. However, it should be noted that other software may perform differently than the ones analysed in this research.

We ran each tool with the default analysis settings since we assumed that this approach is taken by most users. The exceptions involved the exclusion of sequences with only one read count to decrease the likelihood of the presence of sequencing artefacts. Moreover, the miRNA sequence length was set to 18–25 nt, since this is the length range in which most miRNAs fall (Filipowicz et al. 2005; Pawlina et al. 2017; Pawlina-Tyszko et al. 2020). Additionally, longer sequences could increase the number of false positives (Fromm et al. 2022). Nevertheless, it cannot be ruled out that the adjustment of analysis parameters could boost algorithm performance. The performance may also be influenced by the technology of library preparation. The analysed libraries were prepared with ligation-based kits and total RNA input. Libraries based on enriched small RNAs input and/or ligation-free kits utilising poly(A) tailing and template switching could affect the sequencing output differently.

The performed comparison analysis showed differences in the number of known miRNAs and potentially new sequences between individual programs. Each of them also identified a number of unique microRNA sequences (Table 1 and Fig. 1). The qPCR validation confirmed the expression of all selected microRNAs in the tested samples, which proves the high reliability of each of the programs used (Tables 2 and 3). To shed some light on possible functional implications of different performance of the investigated algorithms, we carried out a pathway enrichment analysis for the differentially identified miRNAs. The analysis revealed numerous biological pathways, of which most were common for the tools. On the other hand, similarly to the miRNA profiling results, a number of program-specific pathways were defined (Online Resource 8). This emphasises a possible influence of software choice on the results of in silico functional analysis and, as a result, conclusions drawn. However, further investigation is needed to state if these program-specific microRNAs are always associated with specific biological processes.

Next, we calculated correlation coefficients between the qPCR results and read counts of the examined microRNAs outputted by the analysed algorithms to determine if there is a superior one. In this step, we included qPCR results for additional miRNAs identified by all the algorithms. These served as positive controls not only for qPCR reactions but also for the correlation coefficient calculations since they were identified irrespectively of the ones used in this study program. Significant correlations were identified for two of the six analysed positive controls, by each tested program (Table 4). When it comes to the software unique miRNAs, two of 11 miRDeep2 miRNAs, one of seven sRNAtoolbox-sRNAbench miRNAs, and six of seven UEA sRNA Workbench microRNAs had significant correlation coefficients. Thus, UEA sRNA Workbench showed the highest number of significantly correlated miRNAs. However, since this was true only for its unique miRNAs and was not observed for the positive controls, it is difficult to determine with certainty if it is the result of better algorithm performance or the coincidental choice of better correlating miRNAs for the qPCR validation.

In general, it was noticeable that if a miRNA was detected by at least two programs, its correlation coefficient and *p*-value were similar; i.e. if a microRNA was strongly and significantly correlated, it was so for all the tools (Table 4). This suggests that there may exist some miRNAs which pose a difficulty for identification algorithms. Similar observations were made by Everaert and colleagues on the RNA-seq data. They stated that such a situation takes place for genes that are typically low expressed (Everaert et al. 2017). This remains in agreement with our results; however, it may not be the whole truth for miRNAs. The correlation analysis results obtained in this study for the high-quality and probability miRNAs identified by all the programs and serving as positive controls were significantly correlated for only two of six miRNAs, and significant and insignificant coefficients were noted for sequences with read counts below 100 as well as those above 1000. Thus, other factors could also play substantial roles. One of them may be the afore-mentioned library preparation protocol. It may influence the obtained results introducing library bias associated with the preference of a ligation enzyme for some miRNAs over others (Fuchs et al. 2015). Moreover, primers or assays used for qPCR validation itself may be in some cases the source of variability and discrepancies, as was implicated by the results of qPCR with alternative primers. This may be true especially for unique miRNAs and custom-made assays, which are not wet-lab validated by a manufacturer.

Furthermore, the ubiquitous expression of isomiRs that is microRNA length and sequence variants might be involved. Numerous studies revealed a wide repertoire of isomiRs for many miRNAs. They are very often composed of several dozen different variants with different expression levels (Neilsen et al. 2012; Pawlina et al. 2017), which may vary with statistical significance between tissues, developmental stages, or treatments (Bizuayehu et al. 2012; Fernandez-Valverde et al. 2010; Guo et al. 2016; Loher et al. 2014; Pawlina et al. 2017; Telonis et al. 2015). To shed some light on the potential involvement and significance of these miRNA variants for qPCR validation, we took a closer look at their length and sequence characteristics and the share of the total pool. To this end, we calculated correlation coefficients between the qPCR data and read counts as well as the abundance of separate isomiRs. First, an analysis was performed for the isomiR with the exact same sequence as the used PCR primer assay, assuming the best hybridisation efficiency. This approach caused an increase of correlation coefficients for two of 11 examined miRNAs, namely hsa-miR-100-5p and hsa-miR-433-3p; however, only the hsa-miR-433-3p result was statistically significant (Table 4).

In the next step, we carried out the same analysis for other isomiRs detected for these miRNAs (Fig. 2 and Online Resource 9). This revealed quite a complex picture. First of all, 3′ isomiRs were the most common alteration, which remains in agreement with other research (Loher et al. 2014; Pawlina et al. 2017). We also observed that even variants three nucleotides longer than the used assay can be significantly correlated (hsa-miR-433-3p), so they seem to be quite well tolerated by the PCR assays. Moreover, correlation coefficients may vary for the same length isomiRs but with a different nucleotide added, which may be exemplified by hsa-miR-708-5p for which two-nucleotide 3′ additions were detected (Fig. 2 and Online Resource 9). They were statistically significant with strong and similar correlations except for the “GA” addition, which had the lowest statistics. When it comes to 5′ variants, although they are rarer than 3′ ones and usually shorter (one nucleotide addition or deletion), they were also significantly correlated for many analysed miRNAs (e.g. hsa-miR-433-3p, hsa-miR-100-5p, hsa-miR-223-3p). Furthermore, internal single nucleotide substitutions did not seem to substantially affect PCR performance, since their presence did not rule out significant correlations (e.g. hsa-miR-100-5p and hsa-miR-30c-5p), implying that the assays may bear a level of tolerance for at least some substitutions.

In general, the picture which emerges from this analysis is that the isomiRs composition influence on qPCR results and their correlation with NGS results may be profound and very complex. This may be reflected by hsa-miR-223-3p, for which a moderately significant correlation was established between UEA sRNA Workbench and qPCR results. This miRNA was composed of 13 isomiRs in the analysed samples. The single-isomiR analysis revealed significant correlations with the qPCR results for only three of them (Online Resource 9). The hsa-miR-100-5p analysis showed that the whole miRNA profile correlations between the NGS analysis programs and qPCR results were not significant. However, 21 of 232 detected isomiRs had significant correlation coefficients. On the other hand, hsa-miR-451a was characterised by a strong and significant correlation between the whole miRNA profile NGS results and qPCR results, while 12 of the detected 13 isomiRs were strongly and significantly correlated. The obtained results suggest that if the correlation of the whole miRNA profile NGS results with qPCR results is very strong and statistically significant, the same might be noted for most of the detected isomiRs. When it comes to moderately correlated and less-significant or insignificant miRNAs, there may exist some isomiRs for which significant correlations may be identified if the isomiR profile is broad.

In order to further investigate possible causes of such diversified results for isomiRs, we looked at the number of reads of single isomiRs, assuming that the likelihood of binding of primer sequences to the most abundant variants might be higher. Of the 11 investigated isomiR profiles, the expression level of the most abundant isomiR was strongly and significantly correlated for three miRNAs (hsa-miR-451a, hsa-miR-708-5p, and hsa-miR-375-3p); however, there were other, less-numerous isomiRs with stronger correlations (Fig. 2 and Online Resource 9).

Interestingly, during the analysis of the positive control results, we noticed that two assays examined in samples from two different species (the cow and the pig) performed differently. The correlation analysis results for hsa-miR-375-3p and hsa-miR-708-5p in the cow samples revealed moderately and strongly significant correlations for all the analysed programs (except for UEA sRNA Workbench results for hsa-miR-708-5p). The same assays were not significantly correlated in the pig samples, except for hsa-miR-375-3p UEA sRNA Workbench results and its two separate isomiRs (Fig. 2 and Online Resource 9). Thus, we looked at the isomiR profiles of these miRNAs in the tested samples to see if they may underlie these differences, since isomiR profiles may be even gender and population specific (Loher et al. 2014). However, the profile did not visibly differ in the case of hsa-miR-375-3p and for hsa-miR-708-5p minor differences in the number of identified isomiRs were observed (data not shown). Therefore, it remains to be determined with a higher number of samples and assays if these results are species dependent, tissue dependent, or associated with other factors.

To sum up, factors influencing concordance between miRNA-seq and qPCR data may be very numerous. Some of these may dominate over others and their combination may create unique signatures responsible for correlation results of single miRNAs. Apart from the library protocol bias, the expression of miRNA variants (isomiRs), their abundance, length, sequence composition, and profile characteristics may account in the case of microRNAs. Investigated species could also play a role, although further research is needed to confirm this influence and elucidate its exact nature. The performed analysis implies that the influence of these factors is virtual even for different miRNA-seq identifying algorithms in the case of high-quality and likelihood miRNAs detected by at least two of the analysed programs. This may also be true for other software; however, it remains to be elucidated. Furthermore, additional features that were not assessed here may be of vital importance. Similar observations and conclusions were made for RNA-sequencing analysis workflows also validated with RT-qPCR (Everaert et al. 2017). Finally, we cannot exclude the possibility that miRNAs chosen for the validation favour accurate quantification by NGS or qPCR. The performed analysis would also benefit in the future from an increase in the number of qPCR-quantified miRNAs, single isomiRs, different species and tissues as well as library preparation protocols, used assays, and validation techniques to gain a more comprehensive view.

The most prominent differences between the analysed algorithms were noted for the number of predicted potentially new sequences. sRNAtoolbox-sRNAbench identified 39 while miRDeep2 as many as 2825 novel miRNA candidates. Although these numbers would probably change when fine tuning the algorithm analysis settings, it is rather unlikely that they would even out. All the new sequences unique for the investigated programs, selected for qPCR validation in this study, were confirmed to be expressed, which implies that they may be true novel miRNAs. However, since they constituted only a small portion of all predicted new sequences, this does not guarantee the authenticity of the other ones. Moreover, these results become especially important in the light of the recent analysis performed by Fromm and colleagues who, as a result, postulated that the limits of human miRNA annotation had been met (Fromm et al. 2022). According to the current 22.1 version of the miRBase, 2654 human mature miRNAs are deposited. When it comes to the species investigated here, 1025, 690, and 475 mature miRNAs are currently deposited for the cow, horse, and pig, respectively. These numbers are far from being close to the human data; thus, there still might be a pool of new microRNAs to be discovered in these species, especially in the scarcely investigated tissues and developmental stages. Nevertheless, caution and thorough analysis are advised when identifying novel miRNAs since their repertoire is not limitless.

The results obtained in this study shed some light on the performance of three algorithms for miRNA-seq data analysis and their concordance with RT-qPCR validation. Coenye recently suggested that the added value of qPCR validation is low in the case of high-quality RNA-seq data, highly expressed genes, and those with large differences in expression levels (Coenye 2021). On the other hand, it may be still necessary if these terms are not met. We suggest that in the case of microRNA data, other factors may also be involved and their thorough and detailed characterisation necessitates further, more extensive studies. Whatever the validation decision, miRNA-seq results should be quality controlled and carefully inspected. On the other hand, it should be taken into account that qPCR validation results with moderate concordance with miRNA-seq results are not necessarily the sign of poor NGS quality but may stem from the intrinsic nature of miRNA sequences, the investigated research material, and applied technologies.

## Conclusions

All programs differed slightly in the number of annotated known miRNAs and substantially in the number of predicted new sequences. Of note, each algorithm identified a set of unique miRNAs. All the analysed programs showed a good concordance with RT-qPCR expression data and none of them outperformed the others in terms of the number of confirmed sequences. Interestingly, the highest number of significantly correlated unique miRNAs was identified by UEA sRNA Workbench and the cause of this needs to be additionally confirmed. Careful analysis and interpretation are warranted when assessing RT-qPCR validation results since many different factors and miRNA features may influence them, apart from sequencing quality.

### Supplementary information

Online Resource 1 Sequences of miRNAs for which the custom miRCURY LNA PCR Primers were designed. (DOCX 13 kb)

Online Resource 2 Exemplary histogram showing the length distribution of microRNAs, generated by UEA sRNA Workbench. (PNG 13 kb)

Online Resource 3 Exemplary graphic representation of the secondary structure of a precursor microRNA sequence, generated by UEA sRNA Workbench. (JPG 213 kb)

Online Resource 4 Exemplary stem-loop sequences with their characteristics and mapping details graphically visualized by miRDeep2. (PDF 183 kb)

Online Resource 5 Exemplary plot demonstrating top 10 microRNAs with the highest number of reads identified by sRNAtoolbox-sRNAbench. (PNG 24 kb)

Online Resource 6 Exemplary plot demonstrating the length distribution of a percentage of sequences identified by sRNAtoolbox-sRNAbench. (PNG 8 kb)

Online Resource 7 Results of qPCR validation including biological and technical replicates. (XLSX 28 kb)

Online Resource 8 KEGG pathways significantly enriched in miRNAs identified differentially by the tested programs (FDR≤0.1). (XLSX 21 kb)

Online Resource 9 Results of the correlation analysis obtained for single isomiRs. The sequences of the canonical miRNAs are given as a reference. The assay sequences are bolded, while the sequences of single isomiRs detected are listed below them. Red nucleotides stand for those differing from the reference sequence, while grey cells denote missing nucleotides. “%” stands for the percentage of read counts of individual isomiRs in the whole pool of the identified miRNA variants (calculated on the basis of normalized reads). Positive correlations are indicated with blue bars, while negative correlations with red bars. P-values ≤0.05 are highlighted in yellow. Since the number of identified isomiRs for some miRNAs was very high, only those with p-value ≤0.1 were presented in the file (the exact numbers are provided). (XLSX 34 kb)

## Data Availability

Data used in this study are available in GEO NCBI database under the following accession numbers: GSE87901, GSE148302, and GSE231434.
