# Supplementary material for: Benchmarking of bioinformatics tools for NGS-based microRNA profiling with RT-qPCR method
Source: Funct Integr Genomics. 2023 Nov 30;23(4):347. doi: 10.1007/s10142-023-01276-w (PMC10687144; doi:10.1007/s10142-023-01276-w)
Supplement: Supplementary file 1 — Sequences of miRNAs for which the custom miRCURY LNA PCR Primers were designed. (DOCX 13 kb) [file 10142_2023_1276_MOESM1_ESM.docx]

Benchmarking of bioinformatics tools for NGS-based microRNA profiling with RT-qPCR method

Klaudia Pawlina-Tyszko^1^, Tomasz Szmatoła^1,2^

^1^Department of Animal Molecular Biology, National Research Institute of Animal Production, Krakowska 1 st., 32-083 Balice, Poland

^2^Center for Experimental and Innovative Medicine, University of Agriculture in Krakow, Redzina 1c, 30-248, Krakow, Poland

Corresponding author: Klaudia Pawlina-Tyszko, e-mail: klaudia.pawlina@iz.edu.pl

| **miRCURY PCR assay name** | **miRNA sequence (5’-3’)** |
| --- | --- |
| custom 1 | AACTTTTGCCCCTAGTAACGGACTG |
| custom 13 | GAGGTTACCCGAGCAACTTTGC |
| custom 31 | CACTAGATTGAGAGCTCC |
| custom 47 | TTCCCGCCGGCGTATGCTGCTGTT |

Journal: Functional & Integrative Genomics
