# Supplementary figures and images for: Benchmarking of bioinformatics tools for NGS-based microRNA profiling with RT-qPCR method
Source: Funct Integr Genomics. 2023 Nov 30;23(4):347. doi: 10.1007/s10142-023-01276-w (PMC10687144; doi:10.1007/s10142-023-01276-w)

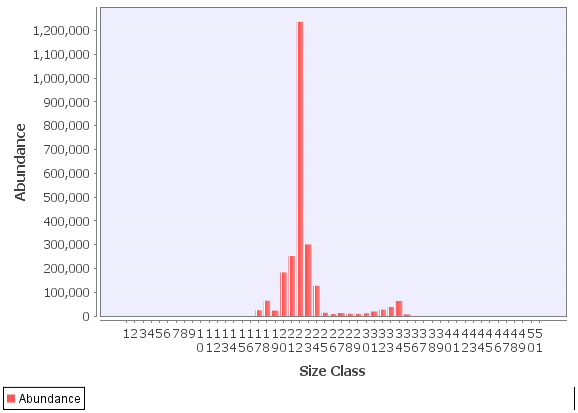

Supplement: Supplementary file 2 — Exemplary histogram showing the length distribution of microRNAs, generated by UEA sRNA Workbench. (PNG 13 kb) [file 10142_2023_1276_MOESM2_ESM.png]

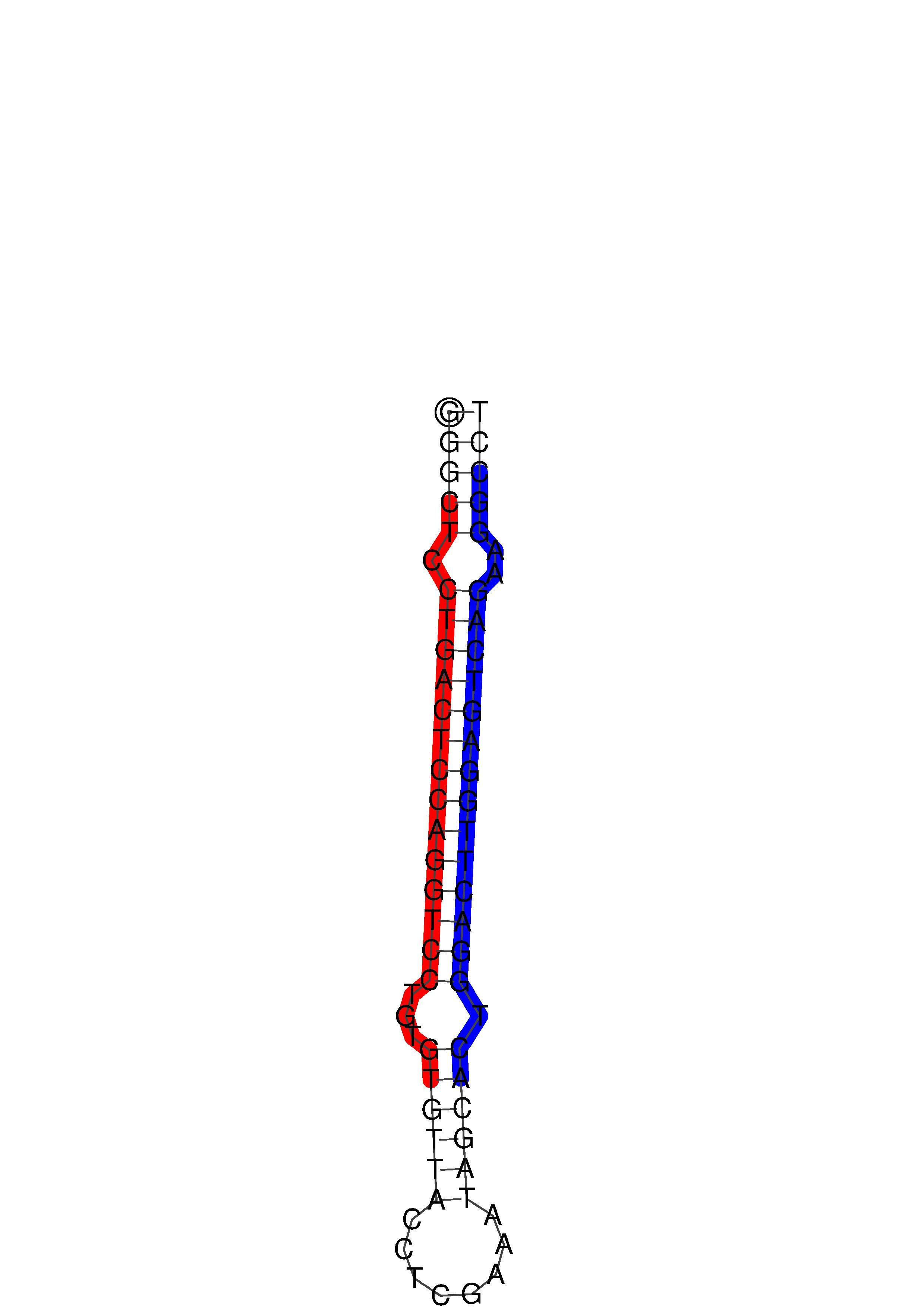

Supplement: Supplementary file 3 — Exemplary graphic representation of the secondary structure of a precursor microRNA sequence, generated by UEA sRNA Workbench. (JPG 213 kb) [file 10142_2023_1276_MOESM3_ESM.jpg]

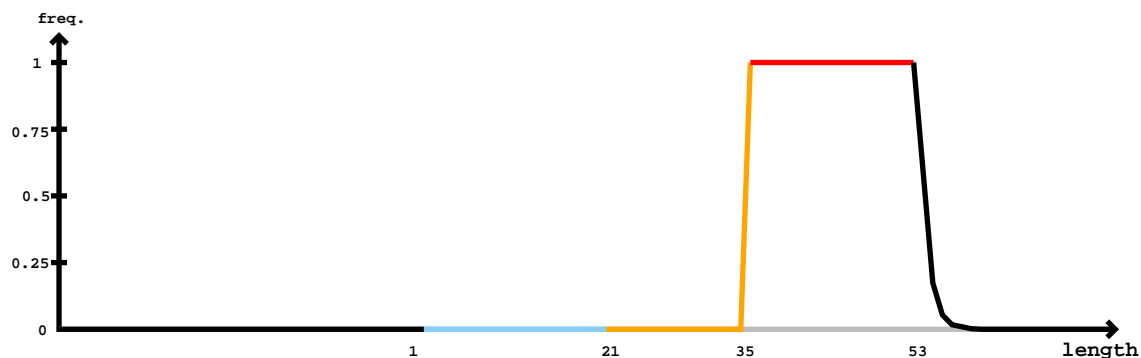

## Mature

[illegible]

Supplement: Supplementary file 4 — Exemplary stem-loop sequences with their characteristics and mapping details graphically visualized by miRDeep2. (PDF 183 kb) [file 10142_2023_1276_MOESM4_ESM.pdf]

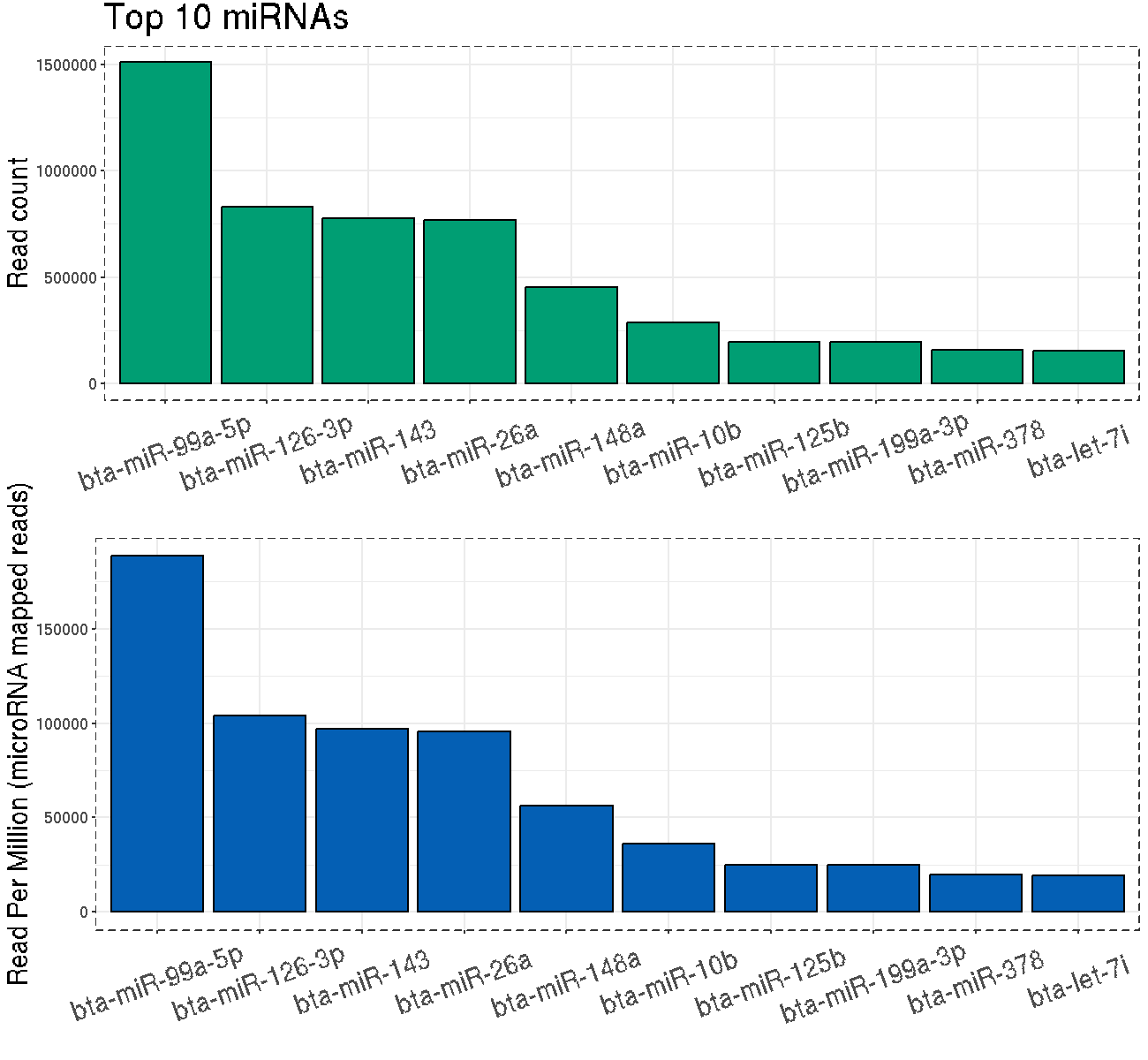

Supplement: Supplementary file 5 — Exemplary plot demonstrating top 10 microRNAs with the highest number of reads identified by sRNAtoolbox-sRNAbench. (PNG 24 kb) [file 10142_2023_1276_MOESM5_ESM.png]

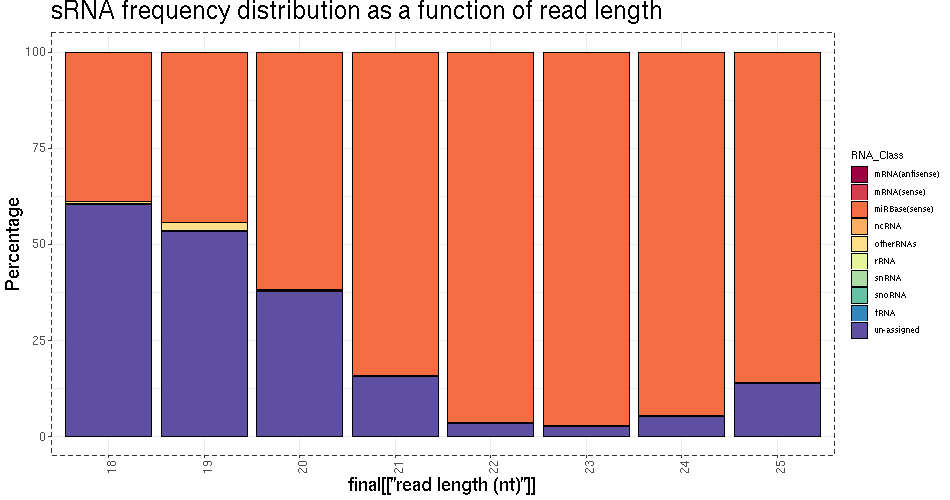

Supplement: Supplementary file 6 — Exemplary plot demonstrating the length distribution of a percentage of sequences identified by sRNAtoolbox-sRNAbench. (PNG 8 kb) [file 10142_2023_1276_MOESM6_ESM.png]
